# Supplementary material for: Discovery of a dual Ras and ARF6 inhibitor from a GPCR endocytosis screen
Source: Nat Commun. 2021 Aug 3;12:4688. doi: 10.1038/s41467-021-24968-y (PMC8333425; doi:10.1038/s41467-021-24968-y)
Supplement: Supplementary file 1 — Supplementary Information [file 41467_2021_24968_MOESM1_ESM.pdf]

Supplementary Information

Discovery of a dual Ras and ARF6 inhibitor from a GPCR endocytosis screen

Jenna Giubilaro, Doris A. Schuetz, Tomasz M. Stepniewski, Yoon Namkung, Etienne Khoury, Monica Lara Marquez, Shirley Campbell, Alexandre Beautrait, Sylvain Armando, Olivier Radresa, Jean Duchaine, Nathalie Lamarche-Vane, Audrey Claing, Jana Selent, Michel Bouvier, Anne Marinier, and Stéphane A. Laporte*

* To whom correspondence should be addressed: Stéphane A. Laporte (stephane.laporte@mcgill.ca)

Supplementary Figures 1-12
Supplementary Table 1

Supplementary Figures

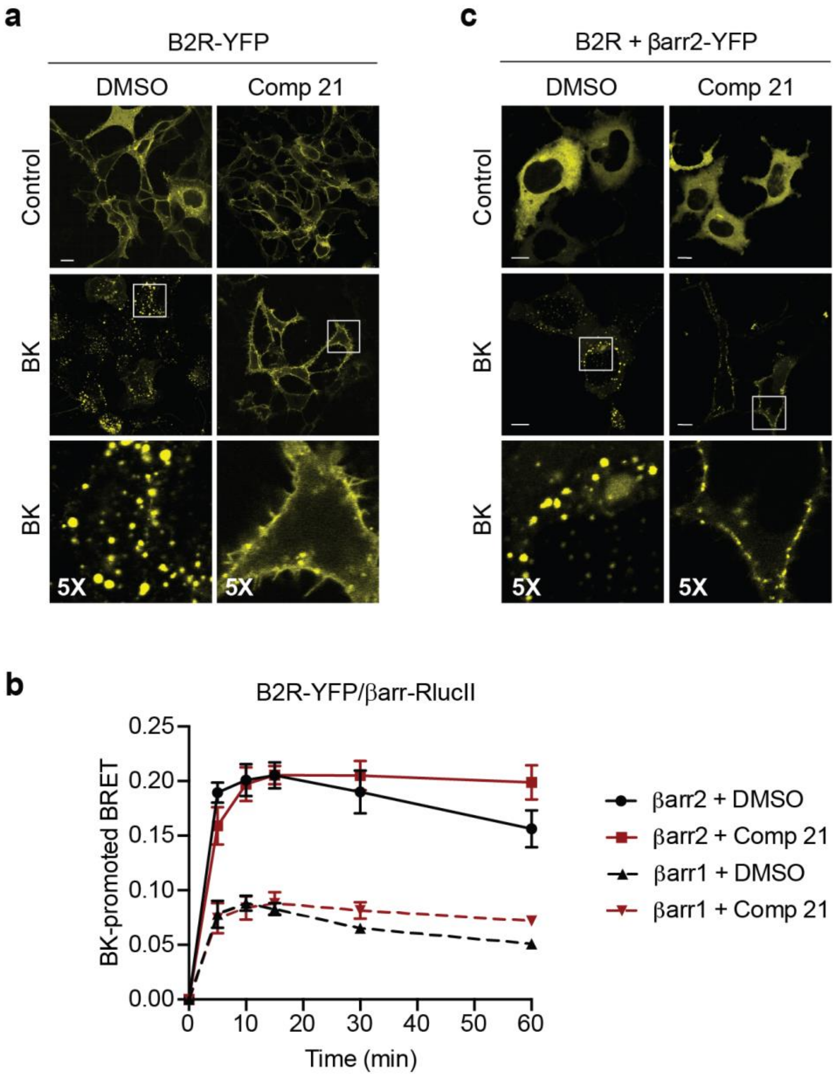

Supplementary Figure 1. Compound 21's effects on the agonist-mediated B2R internalization and β-arrestin1 and β-arrestin2 recruitment to receptors. (a) Confocal microscopy images of YFP-tagged B2R internalization, repeated independently three times with similar results. Scale bar = 10 µm. **(b)** BRET recordings of the recruitment of β-arrestin1 and β-arrestin2 to B2R in absence (DMSO, black triangles and circles, respectively, and lines) or presence of **21** (red triangles and squares, respectively, and lines). BRET responses were quantified as BK-promoted BRET. Data are presented as mean values ± SEM, n = 3 biologically independent experiments performed in triplicate. Source data are provided as a Source Data file. **(c)** Confocal microscopy images of YFP-tagged β-arrestin2 trafficking with B2R, repeated independently three times with similar results. Scale bars = 10 µm. Bottom micrographs are 5X enlargements of the boxed areas. **21** was used at 50 µM.

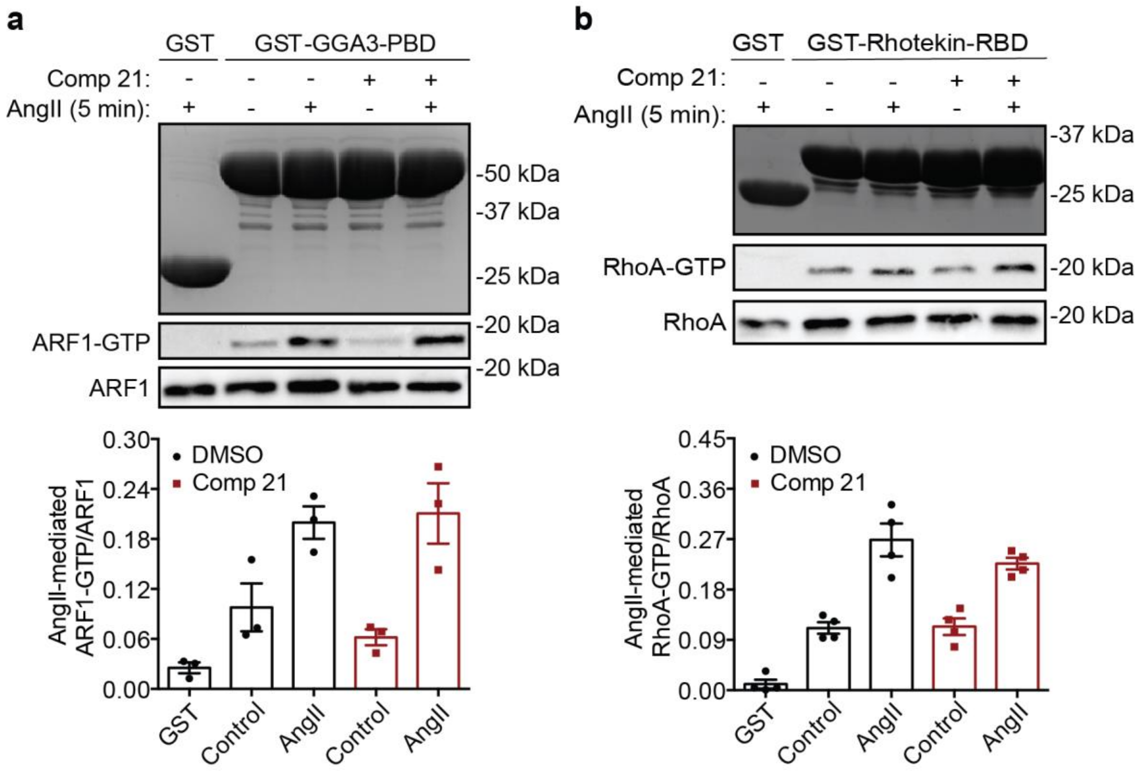

Supplementary Figure 2. Compound 21's effects on ARF1 and RhoA activation. (a) AT1R-mediated HA-ARF1 activation assessed by GST- and GST-GGA3-PBD-coupled to glutathione beads pull-downs in the absence (DMSO, black open bars) or presence of **21** (red open bars). Coomassie of GST and GST-GGA3-PBD proteins, representative western blots and the quantification of HA-ARF1 activation are shown. ARF1 activation was calculated as the amount of HA-ARF1-GTP over total HA-ARF1. Data are presented as mean values ± SEM, n = 3 biologically independent experiments. **(b)** AT1R-mediated Rho activation assessed by GST-Rhotekin-RBD-coupled to glutathione beads pull-downs in the absence (DMSO, black open bars) or presence of **21** (red open bars). Coomassie of GST and GST-Rhotekin-RBD proteins, representative western blots and the quantification of RhoA activation are shown. RhoA activation was calculated as the amount of RhoA-GTP over total RhoA. Data are presented as mean values ± SEM, n = 4 biologically independent experiments. **21** was used at 50 μM. Source data are provided as a Source Data file.

.

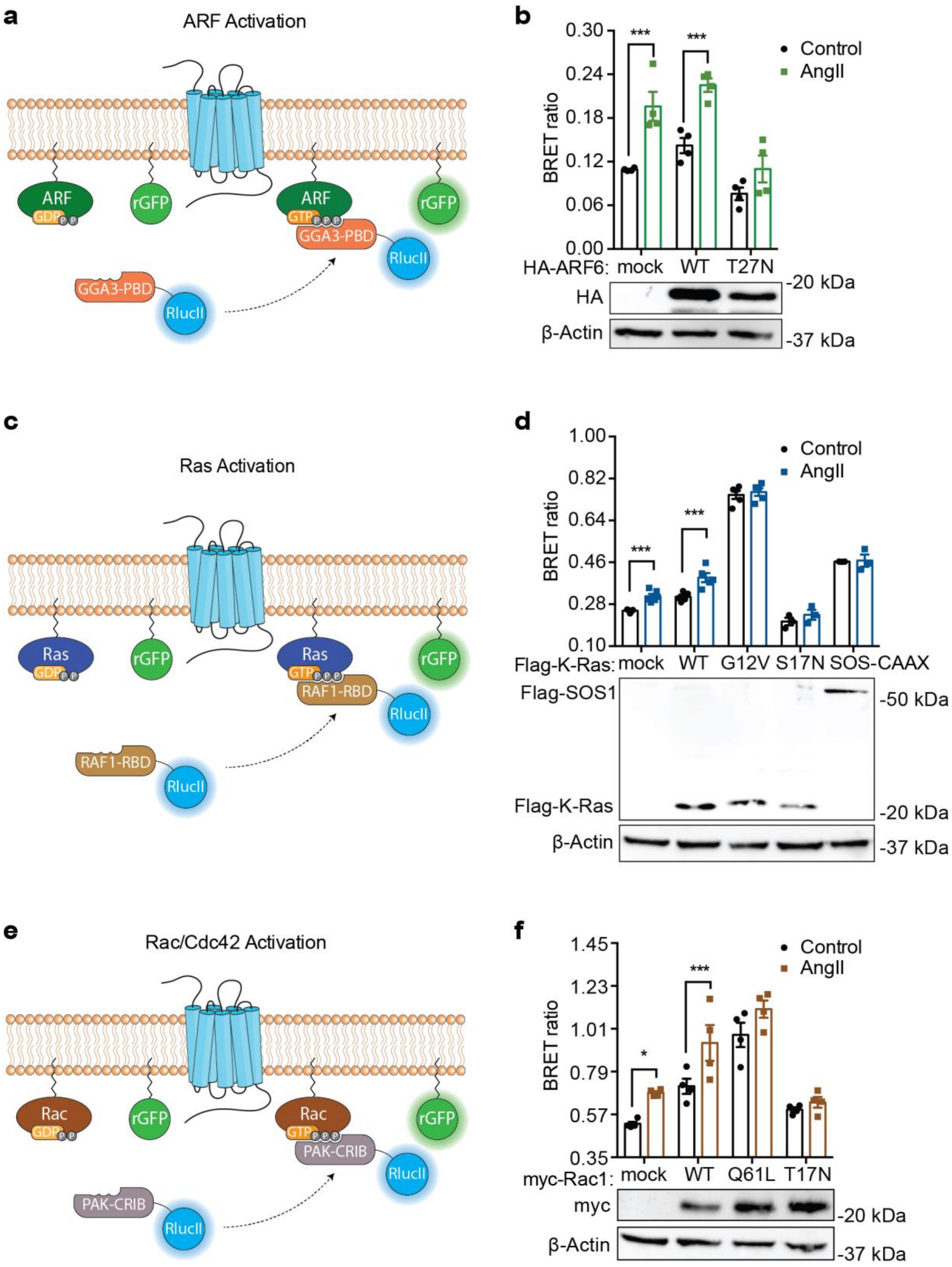

Supplementary Figure 3. Development of ARF, Ras and Rac/Cdc42 BRET sensors. (a, c, e) Schematic diagram of the ARF, Ras and Rac/Cdc42 sensors. **(a)** The recruitment of GGA3-PBD-RlucII to the plasma membrane (rGFP-CAAX) after ARF activation by a receptor generates a bystander BRET signal. **(c)** The recruitment of Raf1-RBD-RlucII to the PM (rGFP-CAAX) after Ras activation by a receptor

generates a bystander BRET signal. **(e)** The recruitment of PAK-CRIB-RlucII to the PM (rGFP-CAAX) after Rac activation by a receptor generates a BRET signal. **(b)** BRET recording of ARF activation by AT1R in HEK293 cells expressing empty vector (mock), HA-ARF6 or HA-ARF6-T27N with AngII (open green bars) or without (control, open black bars). Shown are representative blots of HA-tagged proteins and β-actin as loading controls. **(d)** BRET recording of Ras activation by AT1R in HEK293 cells expressing empty vector (mock), Flag-K-Ras-WT, -G12V, -S17N or Flag-SOS^{cat}-CAAX with AngII (open blue bars) or without (control, open black bars). Shown are representative western blots of Flag-tagged proteins and β-actin as loading controls. **(f)** BRET recording of Rac/Cdc42 activation by AT1R in HEK293 cells expressing empty vector (mock), myc-Rac1-WT, -Q61L, or -T17N with AngII (open brown bars) or without (control, open black bars). Shown are representative western blots of myc-tagged proteins and β-actin as loading controls. Data are presented as mean values ± SEM, at least n = 3 biologically independent experiments performed in triplicate, *$p < 0.05$, **$p < 0.01$, ***$p < 0.005$, two-tailed unpaired Student's *t*-test. Source data are provided as a source data file.

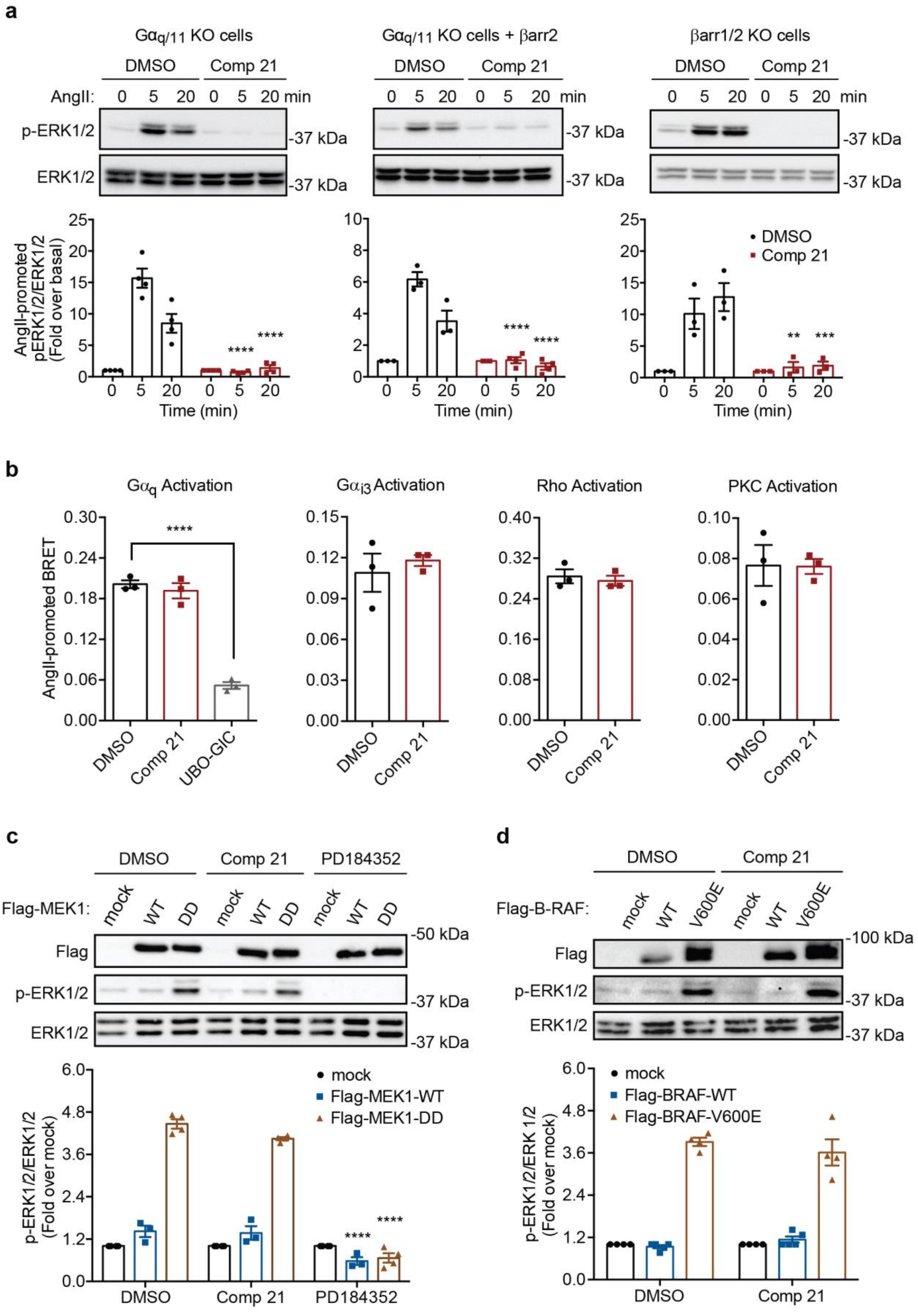
a
Gαq/11 KO cells
Gαq/11 KO cells + βarr2
βarr1/2 KO cells
DMSO
Comp 21
AngII: 0 5 20 0 5 20 min
p-ERK1/2
ERK1/2
-37 kDa
AngII-promoted pERK1/2/ERK1/2 (Fold over basal)
Time (min)
DMSO
Comp 21
b
Gαq Activation
Gαi3 Activation
Rho Activation
PKC Activation
AngII-promoted BRET
UBO-QIC
c
DMSO
Comp 21
PD184352
Flag-MEK1: mock WT DD
Flag
-50 kDa
mock
Flag-MEK1-WT
Flag-MEK1-DD
p-ERK1/2/ERK1/2 (Fold over mock)
d
Flag-B-RAF: mock WT V600E
-100 kDa
Flag-BRAF-WT
Flag-BRAF-V600E
p-ERK1/2/ERK 1/2 (Fold over mock)

Supplementary Figure 4. Compound 21's effects on G proteins and upstream kinases of MAPK pathway. (a) Western blots of AT1R-mediated ERK1/2 activation in the absence of $G\alpha_{q/11}$ (left and middle panels) or β-arrestin1/2 (right panel). $G\alpha_{q/11}$ KO cells were transfected with AT1R alone (left) or along with β-arrestin2 (middle). β-arr1/2 KO cells were transfected with AT1R. Cells were treated with **21** (open red bars) or without (DMSO, open black bars). Data were quantified as p-ERK1/2 over ERK1/2 and normalized as fold over basal (0 min) and compared to DMSO. Data are presented as mean values ± SEM, n = 3 (β-arr1/2 KO cells) and n = 4 ($G\alpha_{q/11}$ KO cells and $G\alpha_{q/11}$ KO cells + β-arr2) biologically independent experiments, $^{**}p < 0.01$, $^{***}p < 0.001$, $^{****}p < 0.0001$, two-way ANOVA with Bonferroni correction. **(b)** AT1R-mediated $G\alpha_q$, $G\alpha_{i3}$, Rho and PKC activation in the absence (DMSO, open black bars) or presence of **21** (open red bars). Cells were transfected with AT1R and corresponding BRET sensors. UBO-QIC (open grey bar): $G\alpha_q$ inhibitor (1 μM). BRET responses were quantified as AngII-promoted BRET and plotted as mean values ± SEM, n = 3 biologically independent experiments performed in triplicate, $^{****}p < 0.0001$, one-way ANOVA with Dunnett's test. **(c)** Western blots of MEK1- and **(d)** BRAF-induced ERK1/2 phosphorylation in cells transfected with Flag-MEK1-WT (open blue bars), Flag-MEK1-DD (open brown bars), Flag-BRAF-WT (open blue bars) or Flag-BRAF-V600E (open brown bars). Western blots of Flag-MEK1 and Flag-BRAF are used as a loading control. PD184352: MEK1/2 inhibitor (10 μM). Data were quantified as p-ERK1/2 over ERK1/2, normalized as fold over mock and compared to DMSO. Data are presented as mean values ± SEM, at least n = 3 biologically independent experiments, $^{***}p = 0.0006$, $^{****}p < 0.0001$, two-way ANOVA with Bonferroni correction. **21** used at 50 μM. Source data are provided as a source data file.

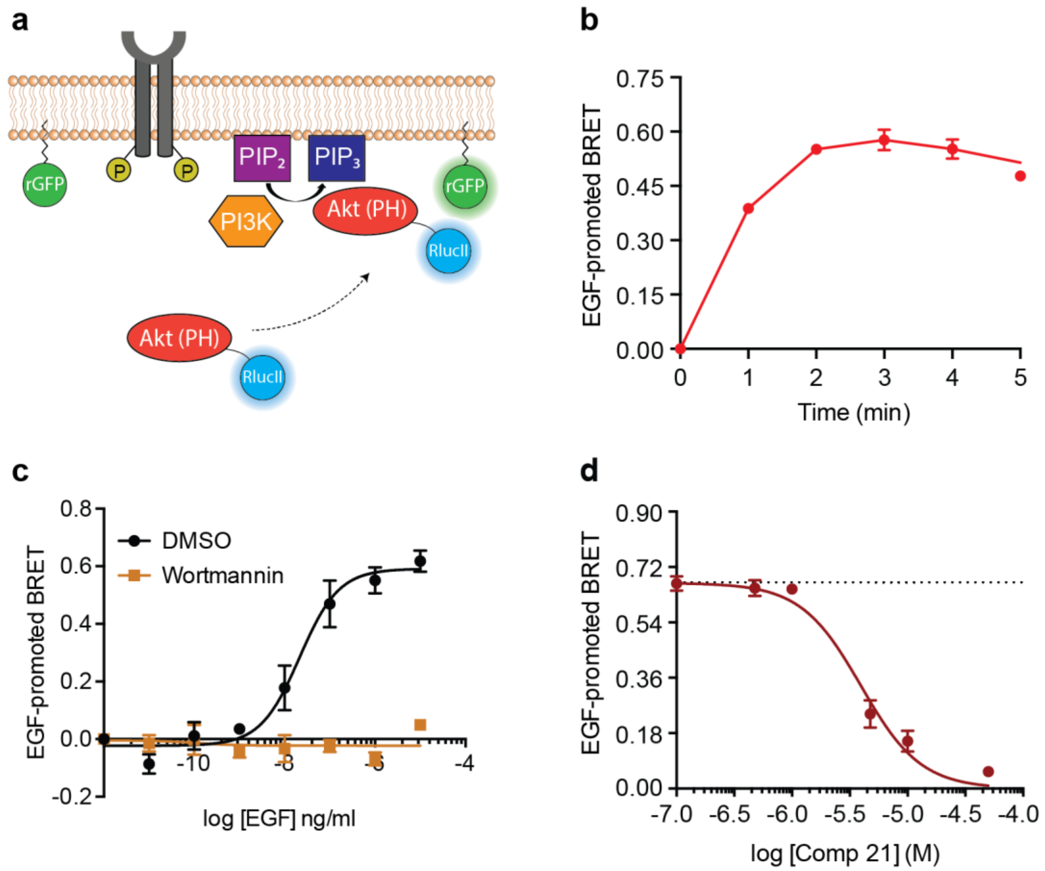

Supplementary Figure 5. Generation and validation of PI3K/Akt BRET sensor. (a) Schematic diagram of PI3K/Akt BRET sensor. Upon activation of PI3K by a receptor (RTK), the recruitment of the RlucII-tagged PH domain of Akt (Akt (PH)-RlucII) to PIP3 at the plasma membrane (rGFP-CAAX) generates a bystander BRET signal. **(b)** BRET recording of the kinetics of PI3K/Akt activation in HEK293 cells expressing Akt (PH)-RlucII and rGFP-CAAX and stimulated with EGF for indicated times. Data were quantified as EGF-promoted BRET and are presented as mean values ± SEM, n = 3 biologically independent experiments performed in triplicate. **(c)** Validation of the PI3K/Akt BRET sensor using Wortmannin (PI3K inhibitor, 200 nM, closed orange squares and line). Data were quantified as EGF-promoted BRET and are presented as mean values ± SEM, at least n = 3 biologically independent experiments performed in triplicate. **(d)** The effects of **21** (50 μM) on PI3K/Akt BRET sensor activation by EGFR. BRET responses were quantified as EGF-promoted BRET, compared to DMSO (dotted line). Data are presented as mean values ± SEM, n = 3 biologically independent experiments performed in triplicate. Source data are provided as a source data file.

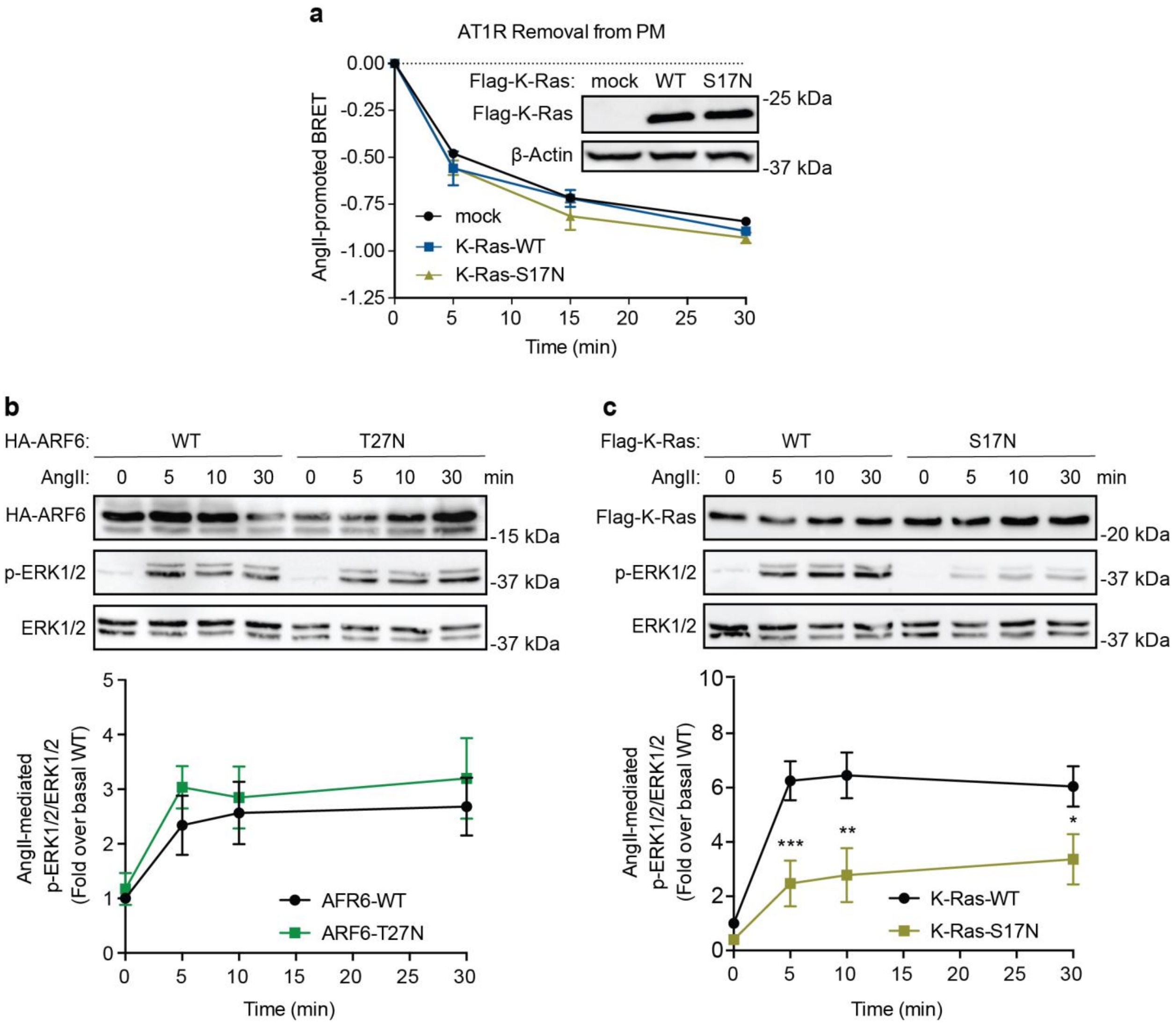

Supplementary Figure 6. The effects of Ras and/or ARF6 on receptor internalization and MAPK activation. (a) BRET assessment of the kinetics of AT1R removal from the plasma membrane in cells transfected with empty vector (mock, closed black circles and line), Flag-K-Ras-WT (closed blue squares and line) or Flag-K-Ras-S17N (closed olive green triangles and line). BRET responses were quantified as AngII-promoted BRET and are presented as mean values ± SEM, n = 2 (mock) and n = 3 (Flag-K-Ras-WT and Flag-K-Ras-S17N) biologically independent experiments performed in triplicate. Western blots of Flag-tagged protein and β-actin as a loading control. **(b, c)** Western blots of AT1R-mediated ERK1/2 activation kinetics in cells transfected with AT1R and **(b)** HA-ARF6-WT (closed black circles and line) or -T27N (closed green squares and line), or **(c)** Flag-K-Ras-WT (closed black circles and line) or -S17N (closed olive green squares and line). Western blots of HA-ARF6 and Flag-K-Ras are used as a loading control. Data were quantified as p-ERK1/2 over ERK1/2, normalized as fold over basal and are presented mean values ± SEM, n = 5 biologically independent experiments, *p = 0.0165, **p = 0.00158, ***p = 0.00117, two-tailed unpaired Student's *t*-test. Source data are provided as a source data file.

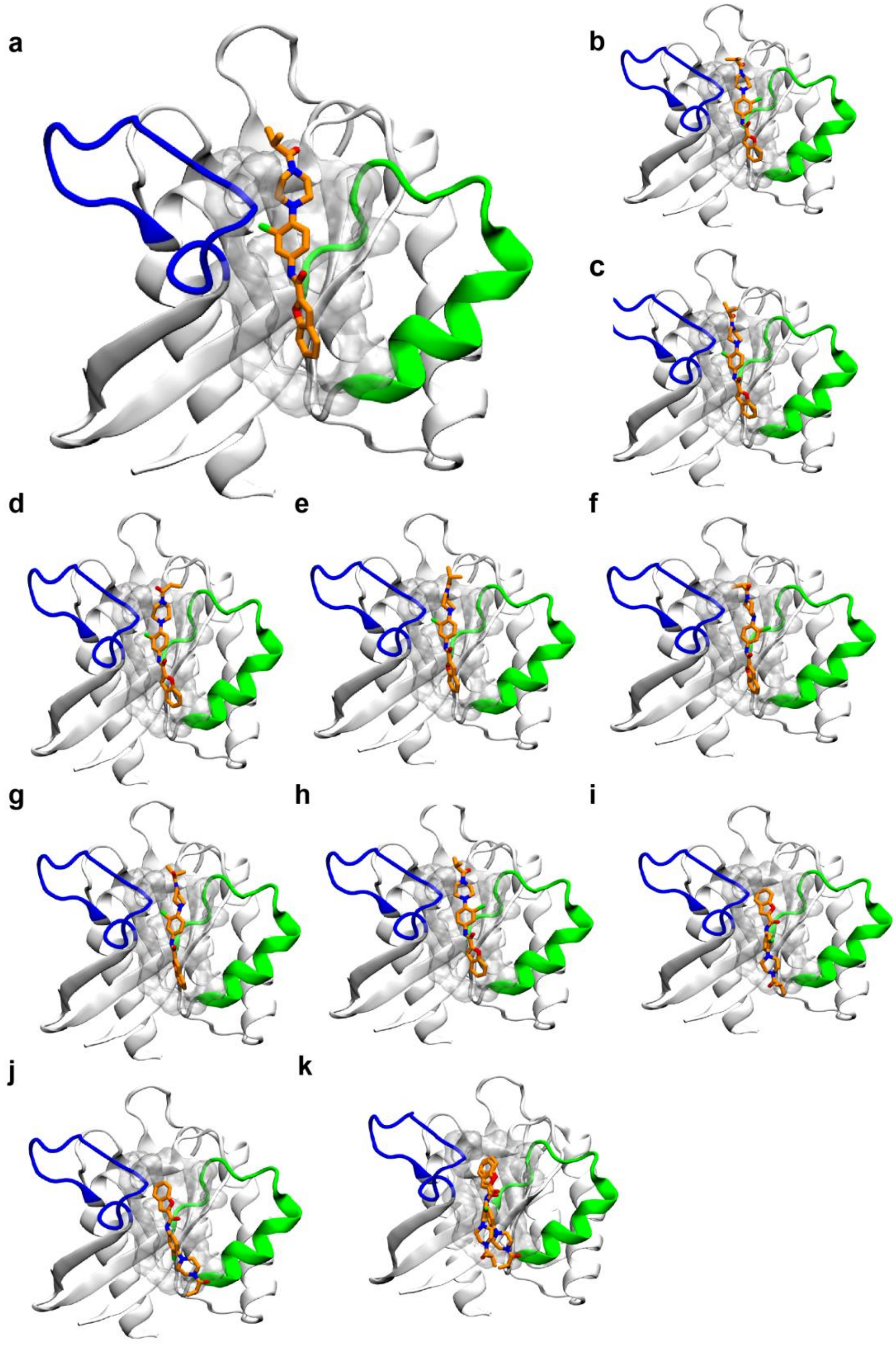

Supplementary Figure 7. Docking poses retrieved from flexible docking of Rasarfin into the Ras-SOS binding groove. (a) Best scored docking pose of Rasarfin (orange licorice) docked in between switch I (blue) and switch II (green) of Ras (white ribbons, transparent surface of binding site shown). **(b-j)** Docking poses 2 – 10. **(k)** Cluster of ligands showing inverted binding mode inside the binding groove (pose 9 and 10 of the docking run).

Rasarfin

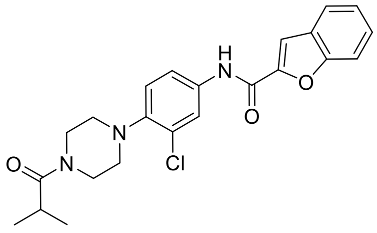

Compound **21.1**

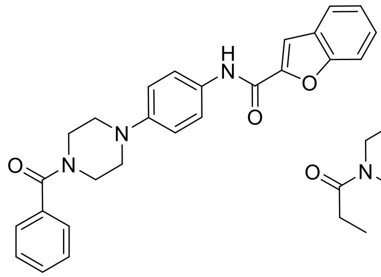

Compound **21.2**

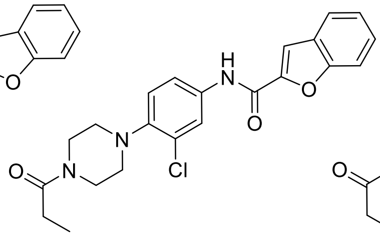

Compound **21.3**

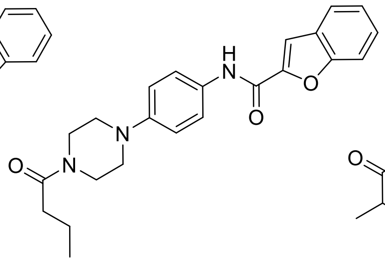

Compound **21.4**

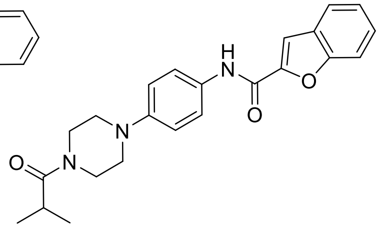

Compound **21.5**

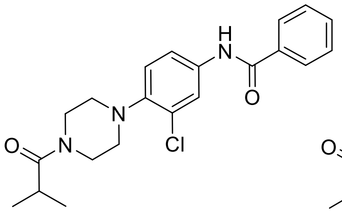

Compound **21.6**

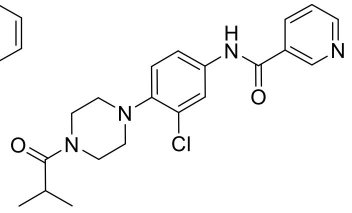

Compound **21.7**

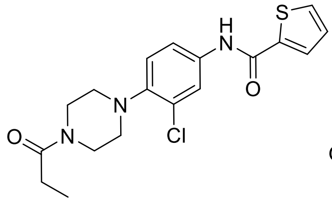

Compound **21.8**

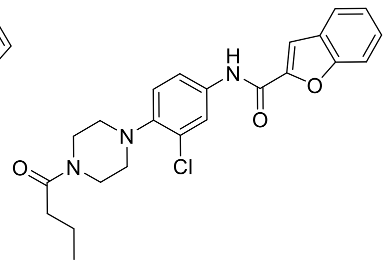

Supplementary Figure 8. Structures of Rasarfin and compounds 21.1-21.8. Shown are the structures of the different analogs of compound **21** (Rasarfin).

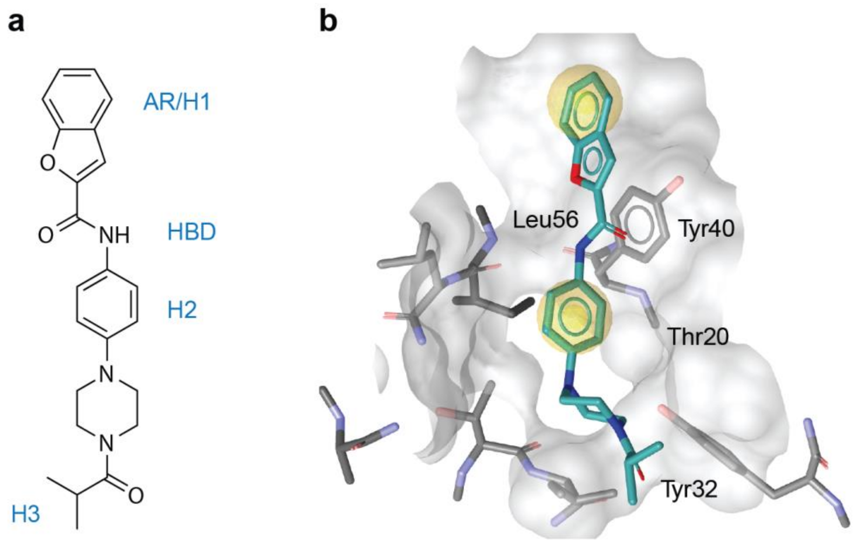

Supplementary Figure 9. Docking model of compound 21.4 onto Ras and its established interactions. (a) 2D structure of **21.4** labeled with established pharmacophoric features (blue). AR: aromatic feature; H1-H3: hydrophobic feature; HBD: hydrogen bond donor. **(b)** Stick representation of **21.4** (turquoise) embedded in the binding pocket of Ras (transparent grey) with interacting Ras residues labelled. Yellow spheres correspond to H1 and H2 of compound **21.4**.

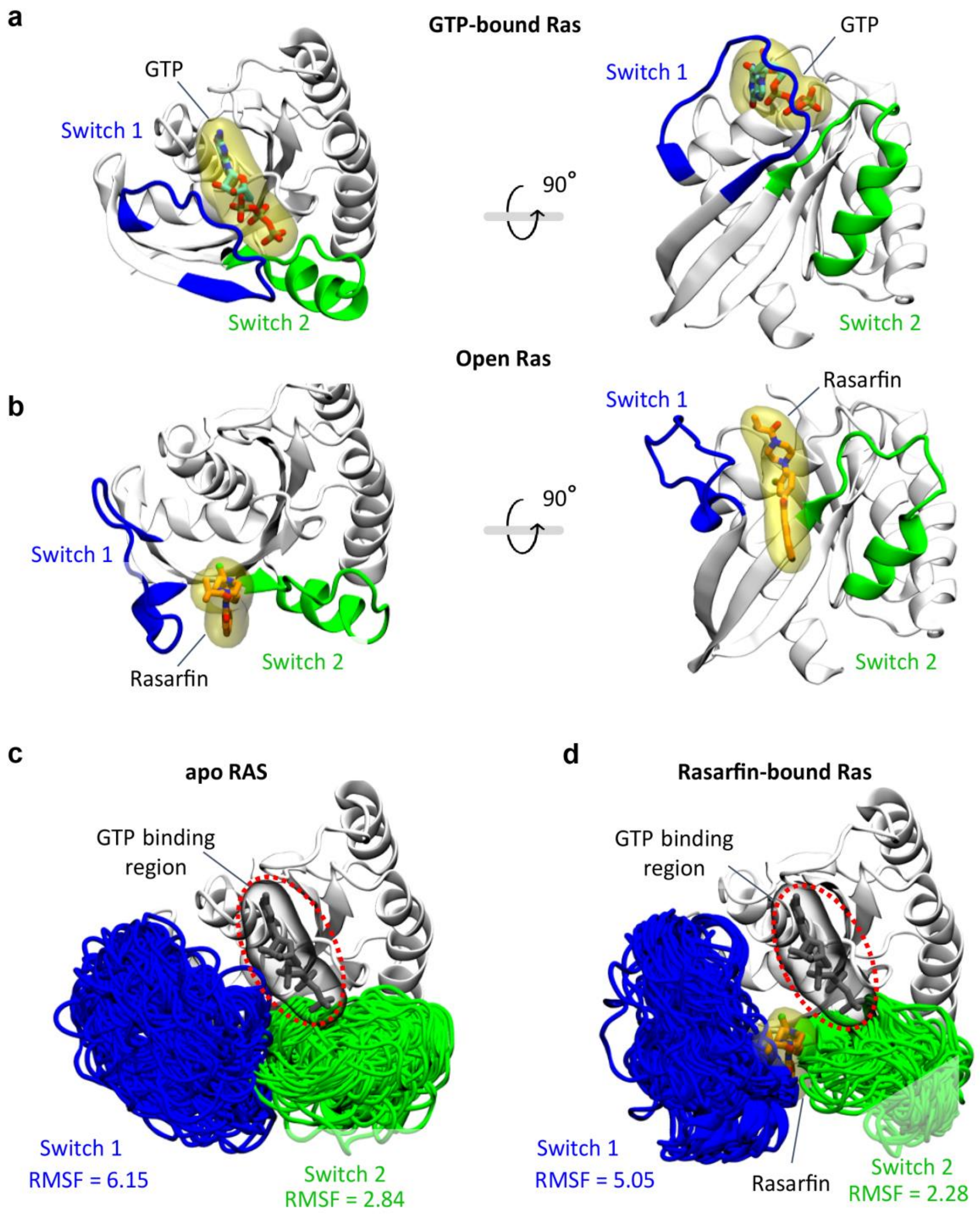

Supplementary Figure 10. Impact of Rasarfin binding on the dynamics of Ras switch I and switch II. (a) Conformations of switch I (blue) and switch II (green) in the closed, GTP-bound (highlighted in yellow) conformation of Ras (PDB: 1QRA). **(b)** Conformations of switch I (blue) and switch II (green) in the open, SOS1-bound conformation of Ras (PDB: 1BKD). **(c)** 250 conformation snapshots of switch I (blue) and switch II (green) extracted from MD simulations of apo state Ras. **(d)** 250 conformation snapshots of switch I (blue) and switch II (green) extracted from MD simulations of Rasarfin-bound Ras. (**c-d**) GTP (grey) binding site (red circle) is highlighted for its positioning in Ras as reference but was not included in the simulation. Structural flexibility of switches is approximated using RMSF, where higher values denote higher flexibility.

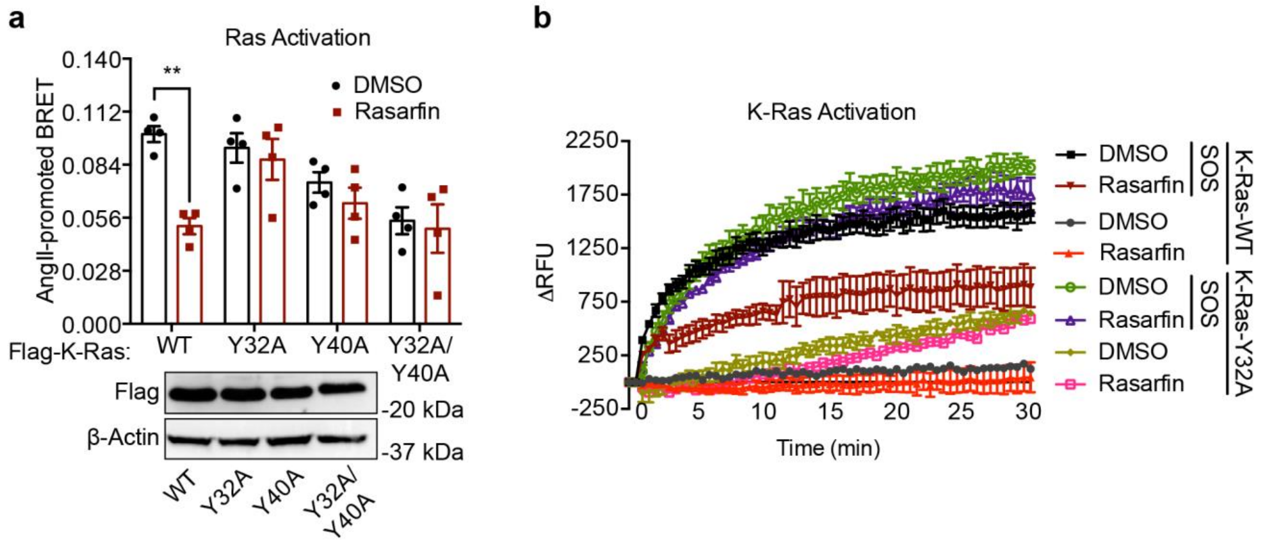

Supplementary Figure 11. Effects of Rasarfin on mutated K-Ras. (a) BRET recording of the effects of **21** (Rasarfin, 50 μM, open red bars) on Ras activation by AT1R in HEK293 cells expressing Flag-K-Ras-WT, -Y32A, -Y40A or -Y32A/Y40A. BRET responses were quantified as AngII-promoted BRET and are presented as mean values ± SEM, n = 4 biologically independent experiments performed in triplicate, **p = 0.0011, two-way ANOVA with Bonferroni correction. Western blots of Flag and β-actin are used as loading controls. **(b)** In vitro kinetics of mant-GTP loading into K-Ras-WT and -Y32A. Purified K-Ras-WT and -Y32A were activated using purified SOS1 and in the presence of DMSO or Rasarfin (50 μM). The relative fluorescence unit (RFU) was measured every 30 sec for 30 min and quantified as the delta RFU. Data are presented as mean values ± SEM, n = 3 biologically independent experiments. Source data are provided as a source data file.

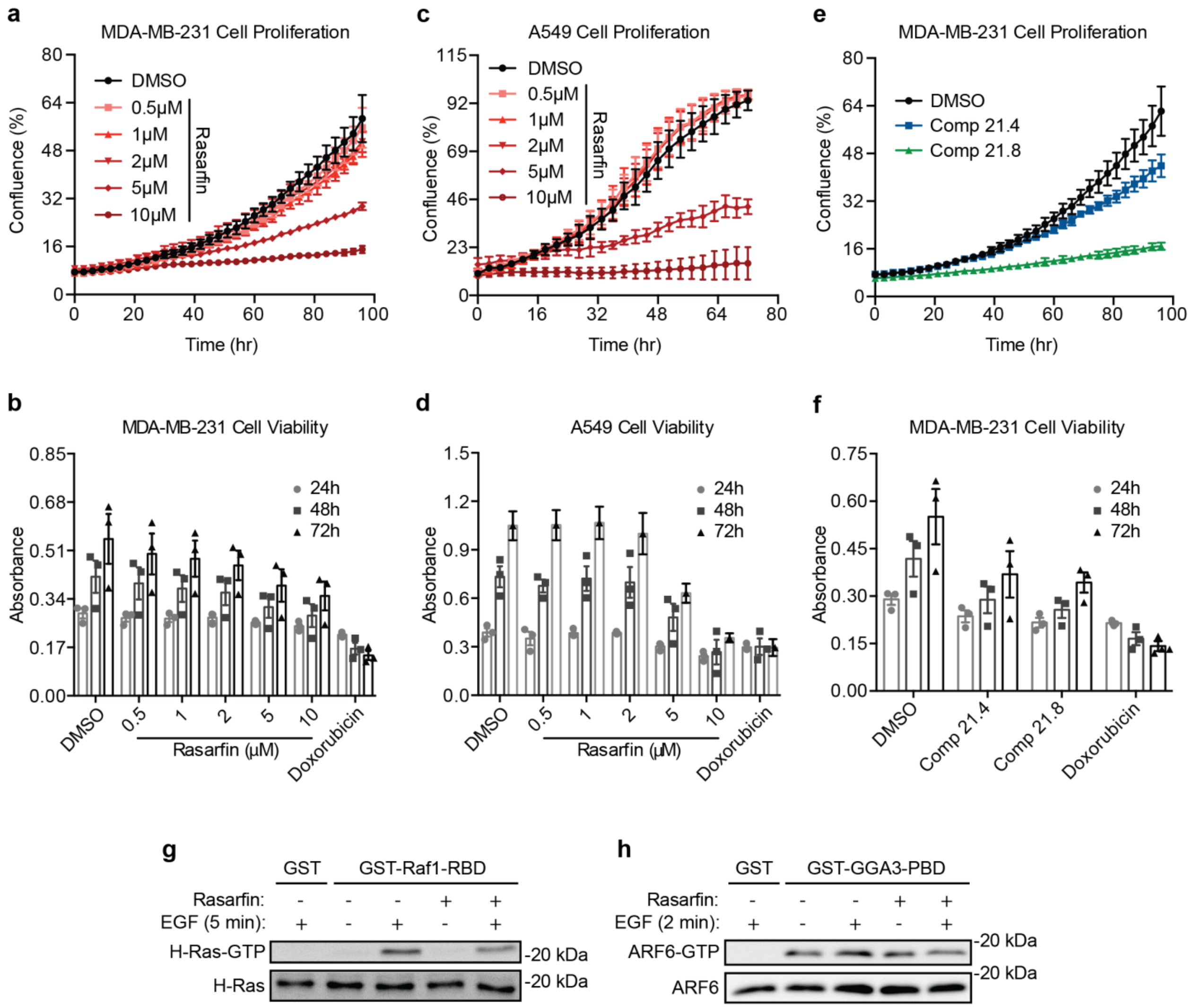

Supplementary Figure 12. Effects of Rasarfin, compounds 21.4, 21.7 and 21.8 on cancer cell proliferation and viability. (a, e) MDA-MB-231 cells were treated with DMSO (black dots and line) or **(a)** various concentrations of Rasarfin (as indicated by different shades of red color), or **(e)** 10 μM of **21.4** (closed blue squares and line) or **21.8** (closed green triangles and line) and cell confluency was measured at the indicated times. Data are presented as percent mean confluency ± SEM, n = 3 biologically independent experiments performed in triplicate. **(b, f)** MDA-MB-231 cells were treated with DMSO, **(b)** various concentrations of Rasarfin (as indicated) or **(f)** 10 μM of **21.4**, **21.8** or Doxorubicin for the indicated times. At each time point, MTT was added and 4 h later, OD (590 nm absorbance) was measured. Data are presented as mean values ± SEM, n = 3 biologically independent experiments performed in triplicate. **(c)** A549 cells were treated with DMSO (black) or various concentrations of Rasarfin (as indicated) and cell confluency was measured at the indicated times. Data are presented as percent mean confluency ± SEM, n = 3 biologically independent experiments performed in triplicate. **(d)** A549 cells were seeded and treated with DMSO, various concentrations of Rasarfin (as indicated) or 10 μM of Doxorubicin for the indicated times. At each time point, MTT was added and 4 h later, OD (590 nm absorbance) was measured. Data are presented as mean values ± SEM, n = 3 biologically independent experiments performed in triplicate. **(g, h)** Western blots of EGFR-mediated H-Ras and ARF6 activation as assessed by GST- and GST-Raf1-RBD- or GST-GGA3-PBD-coupled to glutathione beads pull-downs, respectively, in MDA-MB-231 cells. Experiments were repeated independently three times with similar results. **21** used at 50 μM. Source data are provided as a source data file.

Supplementary Table 1. Sequences of the primers used

Primer Name	Sequence (5'-3')
NheI-GGA3-F	GACCCAAGCTGGCTAGCCACCATGGCGGAGGCGGAAGGGGAAAGCCT
HindIII-GGA3-R	TGGTGGCGGGAAGCTTACCTCCAGAGCCTCCTTCCGAGTCAGGCAGGGTT
RasBD-F	GGCAAACTCACAGATCCTTCTAAGACAAGC
RasBD-R	CATGATCCAGGAAATCTACTTGAAGTTC
NheI-RasBD-F	GAGACCCAAGCTGGCTAGCATGCCTTCTAAGACAAGCAACACTATCC
HindIII-RasBD-R	GTCATGGTGGCGGGAAGCTTGAAATCTACTTGAAGTTCTTCTCCA
PAK1-Crib-F	CCGATCCATTTTACCTGGAGATA
PAK1-Crib-R	GACACAGCCTTCACATTCAAGG
KpnI-PAK1-Crib-F	AGCTGGCTAGCGCCGGTACCGCCACCATGAAAGAGCGGCCAGAGATTTC
AgeI- PAK1-Crib-R1	ATATCGATGGCGCGCCCACCGGTAGCTGACTTATCTGTAAAGCTCAT
AKT-F	TCGGGCACCATGAGCGACGTGGCTATTG
AKT-PH-R	CTTCTTGAGGATCTTCATGGCGTAGTAGCG
NheI-AKT(PH)-F	ACCCAAGCTGGCTAGCGGCACCATGAGCGACGTGGCTATT
HindIII-AKT-PH-R2	TGGTGGCGGGAAGCTTCGAACCTCCACTACCTCCGGAGCT
HRas-F	ATGACGGAATATAAGCTGGTGGTG
HRas-R	TCAGGAGAGCACACACTTGCAGCTCA
BamHI-HRas-F	CTGTTCCAGGGGCCCCTGGGATCCATGACGGAATATAAGCTGGTGGTGG
NotI-HRas-Full-R	AGATCGTCAGTCAGTCACGATGCGGCCGCTCAGGAGAGCACACACTTGCAG CT
KRas-HindIII-F	CCGGACTCTAGCGTTTAAACTTA
KRas-XhoI-R	GGGTTTAAACGGGCCCTCTAGA
KRas-S17N-F	CGTAGGCAAGAATGCCTTGACGA
KRas-S17N-R	TCGTCAAGGCATTCTTGCCTACG
KRas-Y32A-F	GGACGAAGCTGATCCAACAATAGAGGATTCC
KRas-Y32A-R	GGAATCCTCTATTGTTGGATCAGCTTCGTCC
KRas-Y40A-F	TGATCCAACAATAGAGGATTCCGCCAGGAAGC
KRas-Y40A-R	GCTTCCTGGCGGAATCCTCTATTGTTGGATCA
BamHI-KRas-F	CTGTTCCAGGGGCCCCTGGGATCCATGACTGAATATAAACTTGTGGTAGTT
NotI-KRas-R	AGATCGTCAGTCAGTCACGATGCGGCCGCTTACATAATTACACACTTTGTCTT TG
