# Supplementary material for: Discovery of a dual Ras and ARF6 inhibitor from a GPCR endocytosis screen
Source: Nat Commun. 2021 Aug 3;12:4688. doi: 10.1038/s41467-021-24968-y (PMC8333425; doi:10.1038/s41467-021-24968-y)
Supplement: Supplementary file 3 — Description of Additional Supplementary Files [file 41467_2021_24968_MOESM3_ESM.pdf]

Description of Additional Supplementary Files

File Name: Supplementary Data 1
Description: Primary HTS screen data with AT1R. List of 115,303 compounds (with assigned internal user number: UM) and their percent inhibition of AT1R internalization compared to DMSO (expressed as positive values), as well as their effects on the GFP and luciferase signals, as assessed by BRET. Highlighted in yellow is Compound 21 (UM0035925). Negative values express percent potentiation of agonist-mediated receptor internalization. Data are from one experiment. Shown are the raw data and represent single values for each compound.

File Name: Supplementary Data 2
Description: Validation of HTS compounds on AT1R and B2R. List of 40 compounds (with assigned internal user number: UM) that acted as inhibitors or activators of agonist mediated AT1R and B2R trafficking to endosomes as assessed by BRET. Highlighted in yellow is Compound 21 (UM0035925). Results are expressed as percent receptor inhibition (positive values), with negative values expressing potentiation of agonist-mediated receptor internalization. Data represent values for one experiment for B2R and two experiments for AT1R, with one replicate each.

File Name: Supplementary Data 3
Description: KINOMEscan results. Compound 21's effect was tested on 384 kinases. Data represent kinases binding to their ligands in presence of 10 µM of 21, as compared to DMSO (100%). Results are from one experiment and represent the average of duplicates. Three kinases (FLT3, KIT, and PDGFRB) out of 384 were more than 90% affected (selectivity score S(10) of 0.008).

File Name: Supplementary Movie 1
Description: MD simulation of Rasarfin bound to Ras. A movie representing spontaneous fluctuation of Rasarfin (orange licorice) during 500 ns of simulation. Rasarfin is bound between switch I and II on Ras (white ribbons). Residues in direct vicinity (cutoff of 3.5 Angstrom from the ligand) are shown in turquoise. Residues are appearing and disappearing due to the movement of the ligand and the protein. The trajectory has been smoothed for better representation.

File Name: Supplementary Movie 2
Description: MD simulation of compound 21.4 bound to Ras. A movie representing spontaneous fluctuation of compound 21.4 (red licorice) during 500 ns of simulation. Compound 21.4 is bound between switch I and II on Ras (white ribbons) and shows spontaneous unbinding. Residues in direct vicinity of the molecule (cutoff of 3.5 Angstrom from the ligand) are shown in turquoise. Residues are appearing and disappearing due to the movement of the ligand and the protein. The trajectory has been smoothed for better representation.
