# Supplementary material for: Discovery of a dual Ras and ARF6 inhibitor from a GPCR endocytosis screen
Source: Nat Commun. 2021 Aug 3;12:4688. doi: 10.1038/s41467-021-24968-y (PMC8333425; doi:10.1038/s41467-021-24968-y)
Supplement: Supplementary file 9 — Reporting Summary [file 41467_2021_24968_MOESM9_ESM.pdf]

nature research

Corresponding author(s): Laporte, Stephane

Last updated by author(s): 06/07/2021



# Reporting Summary

Nature Research wishes to improve the reproducibility of the work that we publish. This form provides structure for consistency and transparency in reporting. For further information on Nature Research policies, see our Editorial Policies and the Editorial Policy Checklist.

## Statistics

For all statistical analyses, confirm that the following items are present in the figure legend, table legend, main text, or Methods section.

| n/a | Confirmed | |
|---|---|---|
| ☐ | ☒ | The exact sample size (*n*) for each experimental group/condition, given as a discrete number and unit of measurement |
| ☐ | ☒ | A statement on whether measurements were taken from distinct samples or whether the same sample was measured repeatedly |
| ☐ | ☒ | The statistical test(s) used AND whether they are one- or two-sided<br>*Only common tests should be described solely by name; describe more complex techniques in the Methods section.* |
| ☐ | ☒ | A description of all covariates tested |
| ☐ | ☒ | A description of any assumptions or corrections, such as tests of normality and adjustment for multiple comparisons |
| ☐ | ☒ | A full description of the statistical parameters including central tendency (e.g. means) or other basic estimates (e.g. regression coefficient) AND variation (e.g. standard deviation) or associated estimates of uncertainty (e.g. confidence intervals) |
| ☐ | ☒ | For null hypothesis testing, the test statistic (e.g. *F*, *t*, *r*) with confidence intervals, effect sizes, degrees of freedom and *P* value noted<br>*Give P values as exact values whenever suitable.* |
| ☒ | ☐ | For Bayesian analysis, information on the choice of priors and Markov chain Monte Carlo settings |
| ☒ | ☐ | For hierarchical and complex designs, identification of the appropriate level for tests and full reporting of outcomes |
| ☒ | ☐ | Estimates of effect sizes (e.g. Cohen's *d*, Pearson's *r*), indicating how they were calculated |

*Our web collection on statistics for biologists contains articles on many of the points above.*

## Software and code

Policy information about availability of computer code

| | |
|---|---|
| Data collection | Imagelab 5. 2 software was used to quantify the digital blots. Computational softwares used were: VMD Version 1.9.3, Chem Draw 19.1, Ligand Scout 4.4.6., Adobe Illustrator 2020. Chemical Database search: SciFinder CAS solution 2021 |
| Data analysis | GraphPad Prism 6, IncuCyte S3 software (2019A) |

For manuscripts utilizing custom algorithms or software that are central to the research but not yet described in published literature, software must be made available to editors and reviewers. We strongly encourage code deposition in a community repository (e.g. GitHub). See the Nature Research guidelines for submitting code & software for further information.

## Data

Policy information about availability of data

All manuscripts must include a data availability statement. This statement should provide the following information, where applicable:

- Accession codes, unique identifiers, or web links for publicly available datasets
- A list of figures that have associated raw data
- A description of any restrictions on data availability

The authors declare that all data supporting the findings of this study are presented within the article and its Supplementary Information files. Data about the nature of identified molecules in HTS are available upon request.

# Field-specific reporting

Please select the one below that is the best fit for your research. If you are not sure, read the appropriate sections before making your selection.

- [x] Life sciences
- [ ] Behavioural & social sciences
- [ ] Ecological, evolutionary & environmental sciences

For a reference copy of the document with all sections, see nature.com/documents/nr-reporting-summary-flat.pdf

# Life sciences study design

All studies must disclose on these points even when the disclosure is negative.

| | |
|---|---|
| Sample size | No statistical methods were used to predetermine sample sizes. All experiments were done at least three independent times, to a maximum of five, and sample sizes are similar to those reported in the literature and previously by us with these kind of assays (Namkung et al., Sci Signal. 2018 Dec 4;11(559):eaat1631; Luttrell et al., Sci Signal. 2018 Sep 25;11(549):eaat7650; Beautrait et al., Nat Commun. 2017 Apr 18;8:15054 and Namkung et al., Nat Commun. 2016 Jul 11;7:12178) . |
| Data exclusions | No data were excluded from our analyses. |
| Replication | We repeated all experiments using at least three biological replicates (unless stated otherwise) over distinct independent experiments, and frequently repeated by at least by 2 experimentalists. The number of biological replicates and independent experiments are reported in the figure legends. |
| Randomization | No randomization was undertaken. However, experiments were independently repeated by other people in the lab. Samples and cells were often differentially plated and/or loaded on gels between experiments and between researchers performing experiments. |
| Blinding | Blinding was not applied, except for the HTS where the nature of the compounds was blinded in the initial analysis of results. All other experiments were independently repeated by other people in the lab and data analyzed in unbiased manner by at least 2 persons. |

# Reporting for specific materials, systems and methods

We require information from authors about some types of materials, experimental systems and methods used in many studies. Here, indicate whether each material, system or method listed is relevant to your study. If you are not sure if a list item applies to your research, read the appropriate section before selecting a response.

## Materials & experimental systems

| n/a | Involved in the study | |
|---|---|---|
| [ ] | [x] | Antibodies |
| [ ] | [x] | Eukaryotic cell lines |
| [x] | [ ] | Palaeontology and archaeology |
| [x] | [ ] | Animals and other organisms |
| [x] | [ ] | Human research participants |
| [x] | [ ] | Clinical data |
| [x] | [ ] | Dual use research of concern |

## Methods

| n/a | Involved in the study | |
|---|---|---|
| [x] | [ ] | ChIP-seq |
| [x] | [ ] | Flow cytometry |
| [x] | [ ] | MRI-based neuroimaging |

## Antibodies

| | |
|---|---|
| Antibodies used | The phospho-p44/42 MAPK (ERK1/2) (Thr202/Tyr204) (E10) (#9106), p44/42 MAPK (ERK1/2) (#9102), phospho-Akt (Thr308) (#9275), Akt (pan) (C67E7) (#4691), Ras (#3965) and RhoA (#2117) antibodies were purchased from Cell Signaling Technology. The anti-HA-Peroxidase (3F10) (#12013819001), anti-FLAG (#F7425) and anti-c-Myc (clone 9E10) (#M4439) antibodies were purchased from Sigma Aldrich. Anti-mouse and anti-rabbit IgG HRP from BioRad. The β-actin (C4) (#sc-47778), H-Ras (#sc-520/259) and ARF6 (#sc-7971) antibodies were from Santa Cruz Biotechnology. |
| Validation | Phospho-p44/42 MAPK (ERK1/2) antibody #9106: Western blot, validated by vendor with purified phosphorylated and unphosphorylated MAP kinases; https://www.cellsignal.com/products/primary-antibodies/phospho-p44-42-mapk-erk1-2-thr202-tyr204-e10-mouse-mab/9106<br>p44/42 MAPK (ERK1/2) antibody #9102: Western blot, validated by vendor with extracts from siRNA depleted Erk1/2 MAPK HeLa cells; https://www.cellsignal.com/products/primary-antibodies/p44-42-mapk-erk1-2-antibody/9102<br>Phospho-Akt antibody #9275: Western blot, validated by vendor with extracts from NIH/3T3 cells, untreated or treated with PDGF; https://www.cellsignal.com/products/primary-antibodies/phospho-akt-thr308-antibody/9275<br>Akt antibody #4691: Western blot, validated by vendor with recombinant Akt1, Akt2 and Akt3 proteins, and extracts from Hela, NIH/3T3, C6 and COS cell lysates. https://www.cellsignal.com/products/primary-antibodies/akt-pan-c67e7-rabbit-mab/4691<br>Ras antibody #3965: Western blot, validated by vendor with extracts from HT-29, PAE, NIH-3T3, AR42J, PC12 and C6 cell lysates; https://www.cellsignal.com/products/primary-antibodies/ras-antibody/3965<br>RhoA antibody #2117: Western blot, validated by vendor with extracts from HeLa, L929, NIH3T3, and HUVEC cell lysates; https:// |

www.cellsignal.com/products/primary-antibodies/rhoa-67b9-rabbit-mab/2117
HA-Peroxidase 3F10 antibody: lWestern blot, validated by vendor using the the sequence peptide YPYDVPDYA and GST-HA; https://www.sigmaaldrich.com/deepweb/assets/sigmaaldrich/product/documents/760/007/12013819001bul.pdf
FLAG antibody #F7425: Immunoprecipitation and Western blot, validated by vendor using FLAG peptide DYKDDDDK and FLAG-BAP fusion protein from E. coli crude lysate; https://www.sigmaaldrich.com/CA/en/product/sigma/f7425
c-Myc clone 9E10 antibody: Validated by vendor for the detection of peptide sequence EQKLISEEDL; https://www.sigmaaldrich.com/CA/en/product/sigma/m4439?gclid=Cj0KCQjw8vqGBhC_ARIsADMSd1Dsx5N1AbaWDwKNiZ349hb0zxw5SRYo2sfs71ytN-__j2j08y7X5nYaAkhtEALw_wcB
IgG HRP antibodies: Validated by vendor; https://www.bio-rad.com/en-ca/sku/1662408edu-secondary-antibody-goat-anti-rabbit-antibody-conjugated-horseradish-peroxidase?ID=1662408EDU
β-actin antibody #sc-47778: validated by vendor in western blots for β-Actin expression in HeLa, NIH/3T3 and KNRK whole cell lysates; https://www.scbt.com/p/beta-actin-antibody-c4?gclid=Cj0KCQjw8vqGBhC_ARIsADMSd1ByPsq1aPDMwY5kmUk7yjGBndxoiuG-_0e7k27MVqQ6Bbayki03QaIaAlwtEALw_wcB
H-Ras antibody #sc-520/259: validated by vendor in western blots using H-Ras expression in K-562, HeLa, Jurkat, NIH/3T3, A-431 and MCF7 cells and whole cell lysates; https://www.scbt.com/p/h-ras-antibody-259
ARF6 antibody #sc-7971: validated by vendor in western blots using ARF6 expression in Hs 181 Tes, SP2/0, EOC 20, C6 and PC-12 cells and whole cell lysates; https://www.scbt.com/p/arf6-antibody-3a-1?gclid=Cj0KCQjw8vqGBhC_ARIsADMSd1BYncF45D3IcmEeK8oDMGaY_HhCdz7OnCYcwM-l-jllcqtnMmjge0EaAo9CEALw_wcB

## Eukaryotic cell lines

Policy information about cell lines

| | |
|---|---|
| Cell line source(s) | HEK293SL cells were derived from originally obtained HEK293 cells form American Type Culture Collection (ATCC), and characterized in Namkung et al., Nat Commun. 2016 Jul 11;7:12178 . MDA-MB-231 cells were originally obtained from ATCC (Cat# HTB-26) and described in Schlienger et al., 2016 Oncotarget 7, 15811-15827 and Boulay et al., 2008 J Biol Chem 283, 36425-36434. A549 lung cancer cells were originally obtained from ATCC (Cat# CRL-7909). |
| Authentication | Cells were not authenticated |
| Mycoplasma contamination | Cells were tested on a regular basis for mycoplasma contamination, and free of contaminations. |
| Commonly misidentified lines (See ICLAC register) | HEK293 cells. No other commonly misidentified cell lines were used in the study. |
